# Web-based COVID-19 risk communication by religious authorities in Uganda: a critical review

**DOI:** 10.11604/pamj.2021.40.63.27550

**Published:** 2021-09-28

**Authors:** Etheldreda Leinyuy Mbivnjo, Ephraim Kisangala, Andrew Marvin Kanyike, Denis Kimbugwe, Tian Okucu Dennis, Justine Nabukeera

**Affiliations:** 1 Biaka University Institute of Buea, Buea, Cameroon,; 2 Kairos Hospital, Kampala, Uganda,; 3 Faculty of Health Sciences, Busitema University, Mbale, Uganda,; 4 Faculty of Medicine Gulu University, Laroo Division, Gulu, Uganda,; 5 Africa Inland Church (AIC) Litein Hospital, Litein, Kenya,; 6 Lynwood Medical Centre, Ntinda, Uganda

**Keywords:** COVID-19, coronavirus, Uganda, religious authorities, risk communication

## Abstract

The objectives of this study were to explore the content of web-based communication on COVID-19 by religious authorities (RAs) in Uganda and to assess the level of integration of the Uganda Ministry of Health (MoH) and World Health Organisation (WHO) COVID-19 risk communication guidelines into the statements released by these RAs. A grey literature review was conducted by searching the websites of intra- and inter-religious bodies for the terms “COVID-19” and “coronavirus”. Thematic analysis was used to assess the content of RA statements which were also mapped to the items of the MoH and WHO statements. Results indicate that RA communications were centred on COVID-19 description and management; the need to adhere to established guidelines; and the adoption of health-protective behaviours, notably, social distancing and avoidance of misinformation. RAs also discussed the effects of COVID-19 and its control measures on the population and spoke against pandemic-aggravated injustices (gender-based violence and embezzlement). The RA messages incorporated the WHO statement to a greater extent than the MoH statement. In conclusion, RAs played a critical role in delivering public health messages in Uganda during the COVID-19 pandemic, a position we believe should be maximized by public health authorities for effective communication during emergencies.

## Introduction

In Uganda, the government precipitously-before the diagnosis of its first case [1] initiated several social distancing measures including lock down and prevention of mass gathering including suspension of religious gatherings [2]. Most of the president´s directives were to be implemented within a few hours after his address [3]. This made information sharing by local authorities and community leaders difficult, as gatherings were not allowed. During this COVID-19 pandemic, the role of religious leaders in communicating and persuading the population to adhere to the ministry of health COVID-19 directives cannot be underestimated. It is generally accepted that religion has a positive impact on the health and behavior of the community and that religious leaders, who are highly respected, can influence their followers into adopting health promoting behavior [4]. For many years, they have held an influential position in the Ugandan society, been a source of dependable information and identified regularly and easily with almost every Ugandan [5]. It is therefore not surprising that more than 99% of Ugandans belong to at least one religious grouping [6].

This ban on religious gatherings limits in-person communication between religious leaders and their congregations. To continue meeting the spiritual needs of the population, the religious authorities must adopt alternative methods of keeping in contact with the people. Today, a handful of churches provide different online packages to their members [7]. According to the Uganda demographic and health survey report, Ugandans also receive information through the mobile telephones, mass media and internet [8]. Even with the general increase in the internet coverage in Uganda [9], little is known about its usage by the religious institutions as a medium of communicating public health information. In addition, there has been a call for holistic care that addresses people´s spiritual needs during the COVID-19 pandemic [10] which provides an additional rationale for this study. The objectives of this review were to explore the content of web-based communication on COVID-19 by religious authorities (RAs) in Uganda and to assess the level of integration of Uganda Ministry of Health (MoH)/national and WHO/international COVID-19 risk communication guidelines into the statements released by these RAs. The following questions were asked: what are RAs communicating on COVID-19 risk through websites in Uganda? Do web-based statements on COVID-19 risk by RAs in Uganda integrate the Uganda Ministry of Health (MoH)/national and WHO/international COVID-19 risk communication guidelines?

## Methods

Grey literature represents any informally published or unpublished material, in print or electronic form that is not controlled by publishers and provides a rich source of evidence (especially for public health practice) for systematic reviews that complements black literature sources [11,12]. In this study, the term grey literature was used to include all literature, data and information identified as grey [11] with a focus on the platform (website) of communication.

**Search strategy:** we obtained the list of licensed churches in Uganda from the website of the Uganda registration services bureau (URSB). We Google searched each listed church for its official website. Then searched for the terms coronavirus and COVID-19 using the search function of each website and also hand searched the news, press, newsletter, media and announcement pages of each website for any statements on COVID-19. We also Google searched the websites of the following intra- and inter-religious bodies for any communications on COVID-19. Uganda joint christian council (UJCC), the inter-religious council of Uganda (IRCU), Uganda muslim supreme council (UMSC), the national alliance of pentecostal and evangelical churches in Uganda (NAPECU). The national fellowship of born-again pentecostal churches of Uganda (NFBPC), The Seventh-Day Adventist Uganda union (SDAUU), Uganda orthodox church, Uganda episcopal conference (Catholic), seventh-day adventist church central Uganda conference, province of the church of Uganda (Anglican), and born again faith in Uganda. These targeted website searches of religious groups were conducted to reduce the probability of not capturing statements released by other authorities not listed on the URSB website. All website and content searches were conducted between 16^th^ of May and 25^th^ of June 2020.

### The criteria for selecting the articles were as outlined below

**Inclusion criteria:** i) statement on COVID-19 by a religious authority (RA); ii) the statement must have been published between December 2019 and June 2020; iii) statement on official letterhead or website of the RA; iv) statement available in English.

**Exclusion criteria:** i) any third-party report on a RA COVID-19 related statement; ii) any statement on COVID-19 released by a RA that is not recognized by the Ugandan government.

**Search outcome and statement selection process:** Denis Kimbugwe and Andrew Marvin Kanyike independently searched the Anglican list of churches for availability of website and COVID-19 content. Tian Okucu Dennis and Justine Nabukeera performed a similar search for the catholic, born again and seventh day lists of churches while Ephraim Kisangala and Etheldreda Leinyuy Mbivnjo did the same for non-denominational places of worship and Orthodox churches. Ephraim Kisangala and Etheldreda Leinyuy Mbivnjo also independently searched for relevant COVID-19 information from the official websites of the aforementioned intra- and inter-religious organizations. During Google searches, the names of churches were inputted as listed on the government website and the first 10 pages of the results (100 items) were screened for the website of the targeted church. In case of discrepancies in search outcomes, Ephraim Kisangala resolved those that arose between Denis Kimbugwe and Andrew Marvin Kanyike, Etheldreda Leinyuy Mbivnjo resolved those found between Tian Okucu Dennis and Justine Nabukeera while Andrew Marvin Kanyike resolved those that arose between Etheldreda Leinyuy Mbivnjo and Ephraim Kisangala. All reviewers bookmarked each religious website to prevent double counting and to easily recognize websites that were shared by more than one RA. Similar bookmarking strategies have been used in grey literature reviews [13]. The titles and body of the retrieved statements were screened for the terms coronavirus and COVID-19 by Etheldreda Leinyuy Mbivnjo and Ephraim Kisangala and selected based on the criteria outlined above. All discussions were held through the zoom video conferencing platform as all authors were working remotely owing to social distancing regulations that were existing at the time this review was done.

**Quality appraisal:** the authority, accuracy, coverage, objective, date, significance (AACODS) checklist [14] was adapted and used to critically appraise the RA statements. The credibility categories which were relevant in assessing these statements were authority (is the individual or organisation responsible for the content clearly identifiable? yes/no), coverage (is there a clear target audience? yes/no), objectivity (is the author´s standpoint clear? yes/no), and date (is the content clearly dated? yes/no. If no, can the date be closely ascertained? yes/no). Only statements that met at least three of these criteria were included for this review. Disagreements between the credibility assessments done by Etheldreda Leinyuy Mbivnjo and Ephraim Kisangala were resolved by Andrew Marvin Kanyike.

**Data extraction and analysis:** to answer the first research question, a thematic analysis of religious statements was independently conducted by Etheldreda Leinyuy Mbivnjo and Ephraim Kisangala to identify key ideas that were communicated. The religious statements were first read, phrases were highlighted and coded by providing short labels that described the highlighted content. Themes were generated by combining several codes with an identifiable pattern and that contained a central idea communicated by the RAs. Notes were compared and discussed between Etheldreda Leinyuy Mbivnjo and Ephraim Kisangala with input from Andrew Marvin Kanyike and Denis Kimbugwe, resulting in the revision, finalization, naming and definition of themes. No software was used in this analysis, everything was done manually. In order to answer the second research question, the contents of COVID-19 risk communication statements released within the first seven days of April by the Ugandan Ministry of Health (MoH) [15], and World Health Organization (WHO) [16] hereafter, referred to as national and international statements respectively, were compared to the statements on COVID-19 risk communicated by RAs in Uganda. Ephraim Kisangala retrieved the national and international guidelines from the MoH and WHO websites respectively. Etheldreda Leinyuy Mbivnjo and Ephraim Kisangala independently read and matched the key points found in each RA statement to each item of the national and international statements and, in case of any discrepancies, consensus was achieved through discussion.

## Current status of knowledge

**Figure 1:**
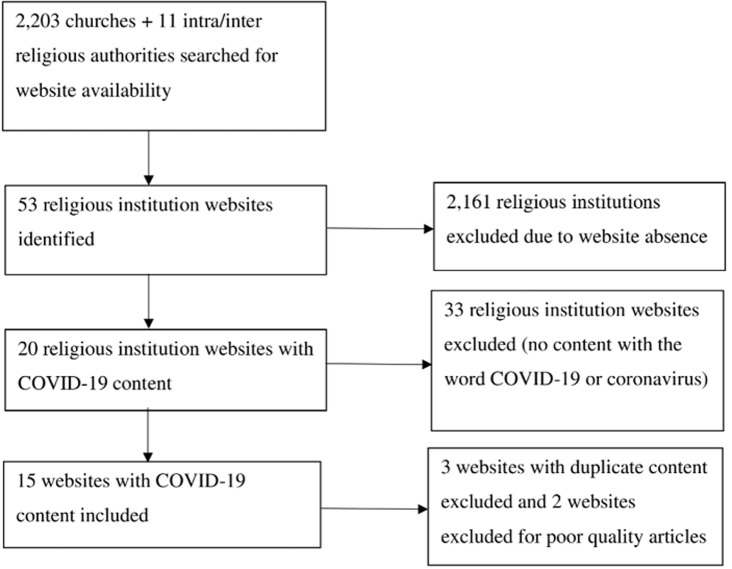
website content selection process

**Search results:** of the 2,214 religious institutions searched for website availability, 53 had websites of which 20 had content on COVID-19. Out of these 20 websites with content on COVID-19, 3 were excluded because the statements were a repetition of what was already published elsewhere. Of the remaining 17 websites, a further 2 were excluded because the target audience and the author´s standpoint or date of publication was not clear. Details of the quality assessment of RA statements can be found in Annex 1. In all, 15 RA websites with COVID-19 content were included for this review and details of the selection process can be found in Figure 1 below.

**Characteristics of selected articles:** most (9/15) RAs released series of statements and two thirds (10/15) of the RAs published at least one message on their website in March 2020. All RA communications were released as a notice or sermon. Most (12/15) RA communications were aimed at their members and leaders and about a quarter (4/15) RAs addressed a section of their message to the government of Uganda and all Ugandans. All RA statements were actionable (what to do or not to do was clearly stated) and in plain English, suitable for lay readers. Each RA was assigned a letter identity (ID) and these IDs ranged from the letter A to O. If the RA released a series of communications, each communication was given a number to show the order of the date in which it was released. For example, A1 was released at an earlier date than A2 and A2 was released before A3 and so on. Details of how the statement IDs were assigned together with other RA statement features can be found in Table 1.

**Table 1:** characteristics of included religious authority statements

Statement ID	Date of publication (dd/mm/year)	Title of document or first line if no title	Type of statement	Target audience	Multiple channel use yes/no	Clarity yes/no	Actionable yes/no
A	21/03/2020(A1), 19/04/2020 (A2), 03/05/2020 (A3), 19/05/2020 (A4), 31/05/2020 (A5)	COVID-19: The unfailing promise of the Lord to protect His people (A1), alive in the peace of Christ, John 20:19-31 (A2), Jesus revealed himself to His disciples at the sea of Tiberia (A3), Midweek fellowship (A4), ''The coming of the Holy Spirit'', Acts 2 (A5)	Sermon (A1, A2, A3 and A5) Notice (A4)	Can't tell (“You”, “Us” and “We” pronouns used)	No	Yes	Yes
B	Can't tell	Message from the Bishop	Notice	People of God, church members, christians, all Ugandans, business people, political leaders	No	Yes	Yes
C	14/03/2020 (C1) 22/03/2020 (C2) 10/04/2020 (C3)	Archbishop kaziimba cautions christians about Coronavirus (C1), provost's communication on Covid-19 (C2), Archbishop's easter message (C3)	Notice (C1, C2 and C3)	Brethren in Christ, christians, clergy, bishops, all Ugandans, security organs, President of Uganda/government, medical staff, media houses	Yes	Yes	Yes
D	10/04/2020 (D1) 24/05/2020 (D2)	The season of Lent, Holy week and the resurrection: parish priest's message (D1), message from Archbishop Cyprian Kizito Lwanga in preparation for the Uganda Martyrs day celebrations (D2)	Sermon (D1), Notice (D2)	Brothers and sisters, People of God	No	Yes	Yes
E	23/03/2020	Pastoral guidelines to all priests, religious and the faithful of Kampala archdiocese as we deal with the threat of Coronavirus	Notice and prayer	People of God	No	Yes	Yes
F	05/04/2020 (F1 & F2) 05/06/2020 (F3)	Bishop Paul ssemogerere's message on COVID-19 (F1), Holy week 2020: special time for heart-to-heart dialogue between God and humanity (F2), Bishop Paul Ssemogerere pilgrimage to the birth place of St. Kizito on Uganda Martyrs Day 2020 (F3)	Notice (F1 and F3), Sermon (F2)	The clergy, the religious, catechists, leaders, people of God in Kasan-Luweroo diocese	No	Yes	Yes
G	03/05/2020	Basilica closed due to COVID-19	Notice	Parishioners and friends	No	Yes	Yes
H	21/03/2020 (H1) 09/04/2020 (H2) 02/05/2020 (H3) 16/05/2020 (H4) 17/05/2020 (H5)	Jesus gives sight and insight (H1), online Holy mass by Mbuya parish during COVID-19 period (H2), The shepherd and shepherds (H3), The spirit of truth amidst uncertainty (H4), Appeal to support the needy (H5)	Notice (H2 and H5) Sermon (H1, H3 and H4)	Brothers and sisters, christians	No	Yes	Yes
I	18/04/2020	Ministry continues during lockdown for COVID-19	Notice	Can't tell	No	Yes	Yes
J	21/03/2020 (J1) 28/03/2020 (J2) 25/04/2020 (J3) 09/05/2020 (J4) 16/05/2020 (J5) 06/06/2020 (J6)	Laetare Sunday! with COVID-19? (J1) Seen the power of the one above all? (J2), Walk with us; stay with us Lord (J3), Jesus the way, teach us to pray (J4), be still and know that God is our God (J5), we rejoice in the Holy Trinity (J6)	Sermon (J1 to J6)	Brethren of Naggulu parish, christians, parishioners	No	Yes	Yes
K	19/03/2020	Guidance by the catholic Bishops of Uganda on the Coronavirus (COVID-19)	Notice	Brothers and sisters, priest, catechists, extraordinary ministers for Holy communion	Yes	Yes	Yes
L	13/03/2020 (L1 is C1) 18/03/2020 (L2) 16/04/2020 (L3) 05/05/2020 (L4) 06/05/2020 (L5)	Pastoral letter from Archbishop Stephen Kaziimba about Coronavirus (L1), Archbishop's guidance on closure of churches (L2), word of encouragement in the Covid-19 crisis (L3), Archbishop's pastoral letter on extension of lockdown (L4), Archbishop's message on the national candlelight event and fight against HIV/AIDS during the COVID-19 pandemic (L5)	Notice	Bishops, clergy, christians, all radio stations, the President, the Government, brothers and sisters in Christ, lay, all Ugandans, development partners, friends of Uganda	No	Yes	Yes
M	13/03/2020 (M1) 13/05/2020 (M2) 22/06/2020 (M3) 22/06/2020 (M4)	Guidelines for religious leaders and faith communities against the spread Corona Virus (M1), urgent appeal for food & other essential commodities (M2), pastoral letter on COVID-19 (M3), project success stories on deepening and amplifying the voices of religious leaders (M4)	Notice	Companies, corporations, the private sector and all/fellow Ugandans, Government of Uganda	Yes	Yes	Yes
N	18/03/2020 (N1) 22/03/2020 (N2) 07/05/2020 (N3) 12/05/2020 (N4) 07/06/2020 (N5)	(Breaking News) church services suspended for a month, the Corona pandemic COVID-19! (N1), understanding the CoronaVirus #COVID-19- explanation by metropolitan Jonah Lwanga of Kampala (N2), a message of hope to all humanity during this COVID-19 situation drawn from the experience of the Myrrh-bearing Women-Rev. Fr. Mutokya Joseph. (N3), arise from your comfort and use it, instead, for better! (Lesson from the Sunday of the paralytic) (N4), pentecost, renewing the living faith (N5)	Notice (N1 and N2) Sermon (N3 to N5)	Brethren (brothers and sisters)	Yes	Yes	Yes
O	Can't tell	The pandemic lockdown	Notice	SDA church members, pastors and evangelists	No	Yes	Yes

### Thematic analysis

**COVID-19 disease and its management:** this theme encompasses the notions of RAs about the origin and description of the disease or virus, its infectiousness, mode of transmission, signs and symptoms, epidemiology, and treatment. The RAs were aware that the virus is zoonotic and was first diagnosed in Wuhan, China. The pandemic was described with metaphors such as the “common enemy” F1, “dark moment” H3, “storm” J2, “battle” J4, “big stone to roll” N3, and “scourge” N4. Religious authorities tracked the evolving epidemiology of the disease in the country by initially stating that “Uganda has not yet registered any case” A1 to when they later declared that “out of all of East Africa, Uganda has the fewest confirmed cases and no deaths” L4. Droplets from an infected person and contact with contaminated surfaces were the modes of transmission discussed by RAs. The signs and symptoms of COVID-19 were generally described as flu-like and the absence of a cure, antiviral agent, and vaccine was also communicated by the RAs.

**Adherence to guidelines:** adherence to guidelines is defined as RAs advice to the people on following the guidance from government, health and religious authorities. Religious authorities urged the people to “adhere to the guidelines given by our civil and religious leaders” J2 and to “embrace the directives given for curbing the spreading of this disease” F1. There was focus on asking the people to follow the directives of the president who was seen as a senior parental figure. The RAs stated that “the president of Uganda, announced the decision of the government to close down all public religious gatherings, he did this as a father who advises his children, we stand with his excellency in this decision”, L2 and that the population should “do as the president has advised, we should not put the Lord our God to the test by neglecting what our elders have advised us to do” C1.

**Health protective and health promoting behaviours:** health protective behaviours refer to the sum total of all information that was provided by the RAs to encourage the adoption of behaviours that reduce the risk of being infected with COVID-19. These health protective behaviours include social distancing (avoiding mass gatherings, staying home, use of non-contact greetings); hand and environmental hygiene; wearing personal protective equipment; practicing cough etiquette; not touching the face; and desisting from misinformation. To enforce social distancing, mass gatherings were cancelled as RAs announced that “all public masses and church programs are suspended until further notice” G, and encouraged unmarried couples to “consider a scientific, Christian wedding” L4 in which only five witnesses and a clergy are to be present. The RAs also stated that “if you are put under self-quarantine,.... do what you are told to do... keep a distance of two meters between you and other people” C1. In addition, RAs advised people to “stay home and stay safe from COVID-19” A4, “avoid handshakes and hugs” B, and adopt non-contact greetings methods such as “waving” C1. Hand hygiene by means of water and soap or alcohol-based hand rub was commonly advised. Religious authorities provided details on how hygiene is to be conducted by stating the duration, “wash your hands with soap and water for at least 20 seconds” C1; effective alcohol concentration in hand rubs and sanitizers, “recommended sanitizer should have above 60% alcohol” K; and using volunteers to model the expected behaviour, “mobilize volunteers at churches and mosques to champion proper hygiene like hand washing” M1. With regards to environmental hygiene, attention was paid to frequent cleaning of “toilets, bath rooms, and other surfaces you may touch water taps, door handles, light switches, cell phones” C1.

In addition, RAs encouraged that personal protective equipment be used when visiting patients: “in the event that you are visiting those sick with the virus, ensure that you use protective wear like mouth and nose masks” M1. The cough etiquette was also recommended by advising on covering the “nose and mouth when coughing and sneezing with a tissue or flexed elbow” N1. There was also a do-not-touch-face caution: “warned against touching faces since the virus can only enter through the eyes, nose and mouth” O. The RAs were cognizant of the existence and impact of misinformation during COVID-19 pandemic. In their communication, they referred to this as a “global misinformation pandemic” H4 and “the post-truth era” J1, warning that such “misinformation or contradicting messages may cause unnecessary panic” H1. They admonished the population against such propaganda: “avoid spreading unsubstantiated information or rumors. Always cross-check any information about coronavirus with health experts, ministry of health, and the website of World Health Organisation” K and recommended that “all faith-based media houses should report approved information on the COVID-19” M1. Health promoting behaviours involved any advice from the RAs that was geared towards the general well-being of the people and not necessarily COVID-19 specific. For example, healthy eating habits were encouraged: “the conference president emphasized a balanced diet, avoiding oils and fatty flesh with sugary foods but encouraged the use of fruits, cereals, greens”O.

**Secondary effects of the pandemic:** secondary effects of the pandemic comprise all the consequences of COVID-19 control measures that slowed down the economy; increased poverty and vulnerability; and disrupted care access or provision for other diseases. The RAs called on the government to take action to revive businesses and likened unemployment and hunger to viruses that came alongside the COVID-19 outbreak. H4 “Since the outbreak of the COVID-19 crisis, the world has faced many other viruses like hunger, unemployment” H4 and “we appeal to the government of Uganda to cushion citizens against the adverse effects of the economic impact of the lock-down” M2. The RAs were concerned about the vulnerable groups who were disproportionately affected by the pandemic mitigation efforts: “*there are many more vulnerable groups such as the elderly, the sick, lactating mothers, refugees and those living from hand to mouth like taxi touts, casual laborers, boda-boda riders among others who cannot fend for themselves during the lock down”* M3. The RAs frowned against the neglect of other pre-existing health problems and looked forward to comprehensive pandemic control measures; ''the pandemic has overshadowed other pandemics like HIV/AIDs which equally needs to be addressed. The council therefore calls for an integrated and inclusive response that addresses all the on-going initiatives on HIV and AIDS, malaria prevention, TB treatment and access to essential sexual and reproductive health services'' L5.

**Injustice during the pandemic:** all unfair practices relating to the misuse of resources, financial exploitation, maltreatment of people and discrimination in connection with the pandemic were defined as injustice. The RAs warned against growing gender-based violence amidst the COVID-19 outbreak. “There has been an increase in domestic and gender-based violence, violence is never acceptable. Never“. The ill treatment of civilians and clergy was also a call for concern “....security organs in the country to enforce the restrictions respectfully; please do not beat your fellow Ugandans as if they were animals” C3 and “....recognize clergy and lay readers as ‘essential employees’.... and should be respected by security personnel when they respond to such calls for prayer” L4. RAs spoke against economic exploitation and warned business owners to refrain from fraudulent practices: “there are some people who try to draw economic benefits from this situation at the expense of others by hiking the prices of items or charging the services which are supposed to be free” F1.

Misappropriation of government resources for the fight against COVID-19 was raised by the RAs, “we were only shocked by our elected members of parliament who creatively allocate themselves a certain percentage instead of putting resources to feed the hungry during lockdown” H3 and “so our representatives´ 20,000,000 COVID-19 money story should not dampen us so much” N4. The RAs were of the opinion that the application of mass gathering cancellations was unfairly implemented since religious institutions were not allowed to open at the time that restrictions in other crowd-pulling avenues such as markets were lessened: ''the powers that be continue to ignore the fact that in markets, car parks, taxi stages... people are not observing social distancing... but for places of worship, we are told that we must hold on... Is COVID-19 selective? Does it bring out its teeth to bite, only in places of worship?'' J6.

**Use of technology:** this refers to the use of technology-enhanced alternatives by RAs to ensure that closure of worship houses does not impair activities such as prayer and offerings. Programs offered by religious institutions were hosted on social media platforms, radio and televisions: “celebrations will be broadcast on various social and electronic media platforms” D2 and “many congregations are offering live-stream services through Facebook and/or YouTube. Save your MBs to watch” L4. For offerings, electronic money transfer methods were used by RAs: “send your gift through mobile money platforms” C2, and “do not neglect tithes and offerings... use online banking... mobile money may be another means” L2.

**Community spirit:** community spirit refers to the advice given and actions taken by the RAs to ensure solidarity in the fight against COVID-19 and that support was given to those in need. This incorporates the call to look after one another improve literacy and support the vulnerable and COVID-19 patients. The RAs encouraged both material and psychological support in terms of food contributions and telephone calls or text messaging to alleviate suffering resulting from the pandemic: “...share the little you have with others” C3 and “...be your brother and sister´s keeper...make sure they have enough food... reach out to them over the phone” L4. For those infected with the new coronavirus, the RAs spoke against stigmatization and called for respect towards its victims: “persons who may have symptoms should not be discriminated against or stigmatized but treated with dignity and love” K. The RAs showed their support for improved literacy by providing parental advice for kids out of school and advocating for programs that will improve bible reading and interpretation skills. ''Parents... encourage them [children] to revise their books...'' B, and ''...develop programs that will equip every Christian to be able to read, interpret, and apply the'' L4.

**Hope versus uncertainty:** this theme represents the mixture of feelings that existed among the RAs with fluctuations between hope in God or a better future and anxiety owing to the COVID-19 related challenges. Though some were hopeful, the RAs had doubts due to the uncertainties of the future. Prayer was seen as key to overcoming the pandemic, “pray to God to find a solution to this epidemic and also finding the right vaccine for it” B and “I also call on the president to declare a weekly day of national prayer“ L2. In addition, the RAs were hopeful that the ongoing difficulties will end, “through challenges, pains, desperation, redundancy, fear, hunger, isolation, poverty... let us keep hope... look forward to life after the crucifixion by COVID-19” D1 and “no motor-vehicle transport, no business, no food relief for many vulnerable citizens, but stay in-door. Is there hope? Certainly yes!” N5. On the other hand, the RAs were anxious about the impact of the COVID-19 pandemic on livelihoods and declared; ''Our lives have been significantly disrupted by the closures of churches, schools, and businesses, the restrictions on movement, and the night time curfew... we have our fears about how to find money for school fees and food; we fear our job security, churches are fearing where their income will come from'' C3. Details on how the codes were developed and grouped into themes can be found in Annex 2.

**Figure 2:**
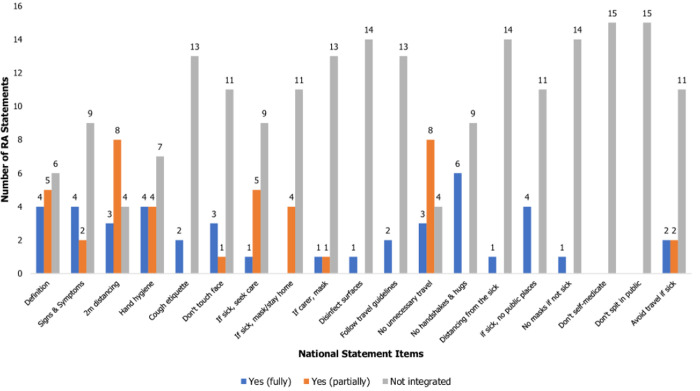
integration of the national (MoH) statement by religious authorities

**Integration of Uganda/National and WHO/International statements into religious authority statements:** an item of either the national or international statements was said to be fully integrated if all parts of it were present in the RA statement, partially integrated if some parts of it were omitted by the RA statement, and not integrated if it was completely missing from the RA statement. Maintaining a reasonable interpersonal distance of at least 2 meters and avoiding unnecessary movements to prevent viral spread, were the most (11/15) widely integrated items from the national statement (Uganda MoH guidelines). However, this integration was mostly partial (8/11) in nature as the 2 metres distance was rarely mentioned by the RAs who mostly focused on asking people to stay home and avoid gatherings of more than 10 people. None of the RAs provided advice on avoiding self-medication and spitting in public as stated by the national guidelines. Details of the level of integration of other national statement items can be found in Figure 2.

**Figure 3:**
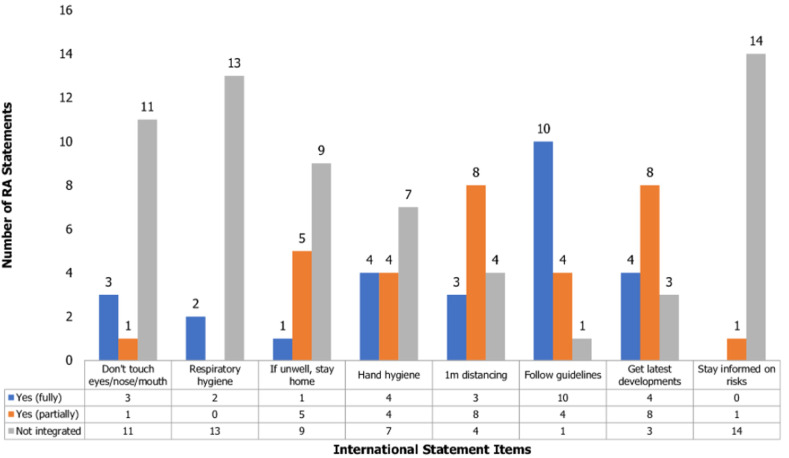
integration of the international (WHO) statement by religious authorities

With respect to the WHO (international) statement, the most commonly integrated item by the RAs was that of following the local and national authority guidelines. Almost all (14/15) RAs mentioned adherence to government guidelines in their statements with most (10/14) of them doing so fully as elaborated by the WHO statement. The least integrated WHO statement was that of staying informed about risks of severe illness as only one RA statement incorporated it partially by warning against infecting “people who are not strong” J4. Details of the level of integration of other WHO statement items can be found in Figure 3. When compared to the MoH statement, the WHO statement was integrated more by the RA statements given that all its items were represented in at least one RA statement as opposed to the MoH statement where two items were completely absent from all RA statements. See Annex 3 and Annex 4 for details on mapping the content of RA messages to the WHO and MoH statement items. Though full integration of most MoH and WHO statements by the RAs was low, the content of RA messages was rich, demonstrating their knowledge and attitude towards the pandemic and the coping strategies that were adopted by religious institutions.

## Discussion

RA communications on COVID-19 started in March 2020, the month in which Uganda went into a nationwide lockdown upon diagnosis of the first COVID-19 cases [2]. All faiths (but for non-denominational churches) as outlined on the URSB website and the Islamic religion were represented in the reviewed websites. Worthy of note is the inclusion of statements from 1 inter-religious body that has a representation from all faiths in Uganda and 4 intra-religious bodies that represent the highest authorities of faiths whose places of worship make up more than 90% of registered churches in Uganda. Though, over half of available RA websites did not provide content on COVID-19, we believe that our sample of 15 websites is qualitatively representative of the views of RAs in Uganda. RAs communication on COVID-19 risks was centred on the following: the origin, description, infectiousness, epidemiology, mode of transmission, signs/symptoms, and management of COVID-19; the need to adhere to established government, health and religious guidelines; and the adoption of health protective and health promoting behaviours. The RAs in Uganda were generally supportive of national efforts to curb the spread of COVID-19 which may have played a role in Uganda´s comparatively low case count, 1,500 cases as of 17^th^ August 2020 [17] which is contrary to what has been documented elsewhere. In the free State and Eastern Cape provinces of South Africa, 80% of all COVID-19 patients were linked to church and burial ceremonies [18]. Similarly, in a rural county in Arkansas (USA), 38% of laboratory confirmed cases and three deaths were attributed to several church events conducted by a pastor and his wife [19]. There are also indications that religious leaders in the USA and South Korea have perpetrated misinformation about COVID-19 in a way that reduces perceived risk and culminates in church-linked clusters of cases [20].

Religious institutions in Uganda made several adjustments to maintain the culture of prayers and offerings when gatherings were restricted. Radio, television and internet-based platforms were used to deliver religious programs while electronic money transfer media were used for offerings. Likewise, evidence shows that religious ceremonies and studies are being held through video conferences as a way of keeping individuals connected to each other thereby making them more resilient during the COVID-19 crisis [21]. In Philippines, online masses have been instituted alongside other initiatives to cater for people´s spiritual needs [10] while the use of online and private prayers are on the rise globally [22]. The effects of COVID-19 and its control measures on the population were discussed alongside the different injustices that occurred during the pandemic. The RAs in Uganda strongly articulated the surge in gender-based violence, another public health crisis that has paralleled previous epidemics and for which warnings have been issued in the ongoing COVID-19 pandemic [23]. It has also been shown that control measures for COVID-19 (lockdown, stay-home, social distancing, self-quarantine) provide conditions favourable for violence against women [24]. This pandemic has also created an opportunity for corruption, crime and conflicts to thrive as many vital processes are bypassed during decision making. As a consequence, exploitation of the vulnerable, corruption and production of substandard goods/services have reportedly increased [25]. Similar cases have been reported in Uganda where top government personnel suspected to have embezzled COVID-19 resources have been arrested [26].

The RAs advised that through a community spirit, the vulnerable and those affected by injustices or COVID-19 should be given material and psychological support while encouraging everyone to be prayerful and hopeful despite the prevailing challenges. Similar gestures were performed by churches in Detroit-USA who provided free food to the elderly, individuals with physical disability, single parents and individuals out of work [27]. There has been formal reportage of a 50% increase in prayer searches on Google demonstrating an increase in spirituality, as individuals pray for the pandemic to cease and find comfort in religious beliefs [28]. In South Africa, the 31^st^ of May 2020 was commissioned for a national prayer to stop the pandemic by the conference of Southern Africa catholic bishops [29] which correlates with the call to pray and have faith in God by RAs in Uganda. This is very important as this COVID-19 crisis has been linked to depression (17.2%) and anxiety (6.3%) within the general population [30]. Generally, full integration of most MoH and WHO statements by the RAs was low but the WHO statement was more incorporated into RA messages than the MoH statement. This maybe because RAs were not provided with national guidelines on what to communicate and thus were left to decide for themselves what to say. As a result, RAs may have used this as an opportunity to discuss equally important issues such as the effect of the pandemic and its control measures; and the adaptations that were made during the pandemic. By so doing, the RAs gave the researchers insight into their knowledge of COVID-19 as a new and highly infectious viral disease. The attitude of the RAs towards the pandemic could also be deciphered as that of encouraging the adoption of preventive measures, supporting the vulnerable, and standing up against all unfair practices. A representation of all WHO statement items in the RA communications indicates that RAs had explored resources beyond national guidelines and used them to inform their speeches.

The findings from this review have several implications for public health communication practice. Firstly, religious institutions are vital community structures through which effective risk communication can be achieved during emergencies as articulated by the president of the 74^th^ general assembly of the United Nations [31]. This is evidenced from the varied audience that the RA statements were addressed to: from religious leaders and worshipers, media houses, healthcare personnel, all Ugandans, development partners, business owners, friends of Uganda, politicians to the government and its leaders including the president. Secondly, the RAs in Uganda on one hand spoke on behalf of the local and national authorities encouraging adherence and adoption of healthy behaviours and on the other hand, they were agents of change advocating for the people and calling-out the government on observed societal ills. Consequently, RAs are channels for communicating risk during emergencies, for transmitting feedback on the communicated messages, and for relaying community concerns to local/national authorities. Also, the RAs are cognizant of their versatility and articulated this by referring to religious leaders as government “essential employees” L4. Therefore, RAs should be an integral part in the design, delivery, monitoring, and evaluation of public health messages. Ministries of health must acknowledge this unique position RAs occupy in the community by supporting them as partners in healthcare delivery for effective risk communication during emergencies. In collaboration with development partners and RAs in Kenya, the government developed a booklet to provide accurate health messages and dispel myths on COVID-19 to congregations [32], an initiative that should be emulated by other countries at the start of epidemics.

This study was not without limitations. To begin with, not all RAs had websites and even those who had, may not have updated them frequently or made communications on COVID-19 through websites. As a result, we may have missed RA communications through other channels (social media, radio and television). It is also possible that we may have omitted non-English RA statements and communications from RAs that have not been licensed by the Ugandan government. In addition, the differential timing of the religious communications and the national/international communications may have influenced our assessment. The rapidly evolving nature of COVID-19 and resultant continuous revision of national/international statements could mean that the low level of full integration observed in the reviewed religious communications can be attributed to the evolving crises and temporal differences in messages released by the RAs and MoH/WHO.

## Conclusion

Religious authorities were quick to release communications at the dawn of the epidemic in Uganda with a focus on the origin, description, infectiousness, epidemiology, mode of transmission, signs/symptoms, and management of COVID-19; adherence to established government, health and religious guidelines; adoption of health protective and health promoting behaviours. To cope with the absence of in-person activities, religious institutions used radio, television, and internet-based platforms to deliver programs while electronic money transfer media were used for offerings. The RAs discussed the effects of COVID-19 and its control measures on the population and spoke against the different injustices that occurred during the pandemic. These RAs also advised that through community spirit, the vulnerable and those affected by injustices or COVID-19 should be given material and psychological support while encouraging everyone to be prayerful and hopeful. Religious authorities incorporated the WHO statement to a greater extent than the MoH statement in their speeches. Overall, RAs played a critical role in delivering public health messages in Uganda, a position we believe should be maximized by public health authorities for effective communication during emergencies.

### What is known about this topic

- Religious leaders are highly respected and hold influential positions in the Ugandan society;
- Over the years, religious leaders have been a source of dependable information helping to relay health messages and influence people to adopt health promoting behaviours.

### What this study adds

- Religious leaders in Uganda are knowledgeable about the origin, infectiousness, mode of transmission and signs/symptoms of COVID-19;
- Religious leaders in Uganda used websites to educate their members and the public about COVID-19 prevention strategies and social cohesion while directing them to use television, radio, social media and electronic money transfer for remote services;
- Religious leaders in Uganda were quick to observe and speak out about the secondary effects of the pandemic and other challenges brought about by the government’s COVID-19 mitigation strategies.

## Annexes

**Annex 1**: quality assessment using an adapted authority, accuracy, coverage, objectivity, date, significance (AACODS) checklist (PDF-62.6 Kb)

**Annex 2**: research question one; what are religious authorities communicating on COVID-19 risk through websites in Uganda? (Thematic analysis) (PDF-142 Kb)

**Annex 3**: research question two; do web-based statements on COVID-19 risk by religious authorities in Uganda integrate the MoH/national COVID-19 risk (PDF-119 Kb)

**Annex 4**: research question two; do web-based statements on COVID-19 risk by religious authorities in Uganda integrate the WHO/international COVID-19 risk communication guidelines? (PDF-85.1 Kb)
